# A Meta-Analysis of Glasgow Prognostic Score and Modified Glasgow Prognostic Score as Biomarkers for Predicting Survival Outcome in Renal Cell Carcinoma

**DOI:** 10.3389/fonc.2020.01541

**Published:** 2020-09-17

**Authors:** Tongyu Tong, Yupeng Guan, Haiyun Xiong, Liling Wang, Jun Pang

**Affiliations:** ^1^Department of Urology, Nephrology and Urology Center, The Seventh Affiliated Hospital, Sun Yat-sen University, Shenzhen, China; ^2^Maternal and Child Health Research Institute, Baoan Maternity & Child Healthcare Hospital, Shenzhen, China

**Keywords:** renal cell carcinoma, Glasgow prognostic score, modified Glasgow prognostic score, biomarkers, meta-analysis

## Abstract

**Purpose:** Accumulative studies suggest the Glasgow prognostic score (GPS) and modified Glasgow prognostic score (mGPS) to be potential biomarkers; however, their prognostic value remains debatable. Our meta-analysis focused on assessing the accurate prognostic value of GPS and mGPS in patients with renal cell carcinoma (RCC) in addition to their effectiveness.

**Methods:** To investigate the relationship between mGPS/GPS and prognostic value in patients with RCC, we performed a comprehensive retrieval of relevant articles from databases such as PubMed, Embase, Web of Science, and Medline up to February 1, 2020. STATA 15.0 software was used to obtain pooled hazard ratios (HRs) and their 95% confidence intervals for survival outcome, including overall survival (OS), recurrence-free survival (RFS), progression-free survival (PFS), and cancer-specific survival (CSS). A formal meta-analysis of these outcomes was performed.

**Results:** In total, 2,691 patients with RCC were enrolled from 15 cohort studies. Higher GPS/mGPS (GPS/mGPS of 2) indicated poorer OS, CSS, PFS, and RFS in patients with RCC. Similarly, medium GPS/mGPS (GPS/mGPS of 1) also had a significant association with poorer OS, CSS, PFS, and RFS but superior than higher GPS/mGPS in these patients.

**Conclusion:** GPS and mGPS are effective biomarkers for predicting prognosis in patients with RCC, and higher GPS and mGPS are closely related to inferior survival outcomes. More randomized controlled trials are needed to investigate the promising value of GPS/mGPS in the future.

## Introduction

There are more than 1.8 million new cancer cases in the USA alone in 2020, and around 4% of which are estimated to be kidney or renal pelvis tumors (1). Renal cell carcinoma (RCC) is the most common solid tumor of the adult kidney, accounting for 87% of all renal malignant tumors (2). Curative intervention via surgical resection (either partial or radical nephrectomy) has been the primary standard treatment for individuals with clinically localized RCC (3). Although most patients with RCC have localized tumors that can be cured with surgery, immunotherapy, and targeted therapies, the long-term patient prognosis is still disappointing. Many patients have suffered disease relapse caused by local recurrence or distant metastases; however, the absence of consensus on the optimal surveillance strategy and prognostic biomarkers is adverse to disease management (4–6). Thus, finding reliable and precise prognostic biomarkers is becoming increasingly important.

The C-reactive protein (CRP)/albumin (Alb) ratio was calculated by dividing the serum CRP level by the serum albumin level. A CRP concentration of >10 mg/l was considered to indicate the presence of systemic inflammatory response. Consequently, elevated preoperative CRP is associated with poor outcomes relating with metastasis and mortality. Professor Forrest first reported the significance of the prognostic value of the combination of elevated CRP levels (>10 mg/l) and hypoalbuminemia (Alb <35 g/l) (7). This prognostic score, which was based on systemic inflammation, was subsequently termed the Glasgow prognostic score (GPS) (8). The resultant GPS (0, 1, or 2) was defined to be calculated as follows: patients with neither of these abnormalities were allocated a score of 0, patients with only one of these biochemical abnormalities were allocated a score of 1, and patients with both an elevated CRP level (>10 mg/l) and hypoalbuminemia (Alb <35 g/l) were allocated a score of 2. Later, this prognostic score was modified and was termed as modified Glasgow prognostic score (mGPS), which was equal to a score of 1 only for an elevated CRP level (9). Consequently, mGPS reflected infrequent cases of hypoalbuminemia without an elevated CRP level and revealed that hypoalbuminemia might not be related to poor survival (9). Compared to the conventional combination of stage and performance status CRP and albumin levels (7), the combination of organismic inflammatory response and albumin levels was found to have a comparable prognostic value. In addition, GPS and mGPS were useful for the prognosis of various solid tumors, including gastrointestinal cancer (10), non-small cell lung cancer (11), colorectal neoplasms (12), urothelial carcinoma (13), as well as RCC (14).

At present, the number of studies revealing the correlation between GPS/mGPS and the survival outcome of RCC is increasing, and the results indicate the prognostic value of GPS/mGPS (15–20). Controversially, several recent systematic reviews and meta-analyses have provided increasing evidence, but the results remain inconclusive. This field is moving rapidly, and under the current circumstance, our aim was to conduct an updated meta-analysis and subgroup analysis, gathering all levels of available evidence. We aimed to provide accurate prognostic information of potential biomarkers for clinical doctors and patients.

## Methods

### Search Strategy

To investigate the relationship between GPS/mGPS and the survival outcome in patients with RCC, we performed a comprehensive search for relevant studies from public online databases, including PubMed, Embase, Web of Science, and Medline, up to April 1, 2020. The following keywords and medical subject headings were used as search terms: renal cell carcinoma or kidney neoplasms and Glasgow prognostic score or modified Glasgow prognostic score or “GPS” and “mGPS.”

### Inclusion and Exclusion Criteria

All the relevant included studies met the following criteria: (1) patients were diagnosed with RCC by histopathological analysis; (2) routine laboratory measurements, including C-reactive protein and albumin, were performed preoperatively in addition to GPS/mGPS, which was graded and recorded before surgery or treatment; (3) endpoints, such as overall survival (OS), cancer-specific survival (CSS), recurrence-free survival (RFS), and progression-free survival (PFS), were explored in cohort studies (OS was defined as the date from treatment to death for any reason; CSS was defined as the date from treatment to death due to cancer or to the last follow-up; RFS was defined as the time between curative treatment and the confirmation of local recurrence and distant metastasis; and PFS was defined as the date from treatment to tumor progression or death); and (4) corresponding data were present in the form of HRs with 95% confidence intervals (CIs) in the articles or relevant survival data were shown in the Kaplan–Meier curves. Duplicate studies, books/chapters, reviews, case series, letters, editorials, abstracts of conferences, animal experiments, and incomplete or erroneous data were excluded.

### Data Extraction

Two investigators (Tongyu Tong and Yupeng Guan) independently analyzed the following data and consulted with a third investigator (Jun Pang) if any controversy existed: (1) study characteristics, including the first authors, published year and study region, sample size, study duration, details of GPS/mGPS, and its respective ratios; (2) patient characteristics, including age, gender, follow-up period, and interventions; (3) information about RCC, including tumor entities, stage, tumor type, and distant metastasis; (4) GPS/mGPS; and (5) outcomes, including OS, CSS, RFS, and PFS. Engauge Digitizer software 4.1 (http://digitizer.sourceforge.net/) was used to digitize and extract the relevant survival data from the Kaplan–Meier curves. In the case of divergences and discrepancies, the subject was referred to a third-party ruling.

### Quality Evaluation

The methodological quality of the observational studies was independently evaluated by two investigators (Haiyun Xiong and Tongyu Tong) according to the Newcastle–Ottawa scale (NOS) quality assessment tool. Each included cohort study was assessed on the basis of the following categories: selection, comparability, and ascertainment of outcome. We considered studies with scores above 6 as high-quality studies and enrolled them. Divergences and discrepancies were resolved through discussion or consultation with a third party.

### Statistical Analysis

STATA 15.0 software (Stata Corporation, College Station, TX, USA) was used to conduct the present meta-analysis. HRs with 95% CIs from all eligible studies were pooled via a meta-analysis to access the survival endpoints. Heterogeneity among the outcomes of the studies included in this meta-analysis was evaluated using Cochran's *Q-* and *I*^2^-tests. An α value equal to 0.1 and a *P* < 0.05 indicated a statistical significance. An *I*^2^ > 50% was considered to have some degrees of heterogeneity existing, which led to the use of the random effects model; otherwise, the fixed effects model was used. Sensitivity analysis was performed by removing single study in sequence at a time to evaluate the robustness of the pooled results. We also explored publication bias through Egger's tests; a *P* > 0.05 indicated negligible potential publication bias.

## Results

### Study Characteristics

**Figure 1:**
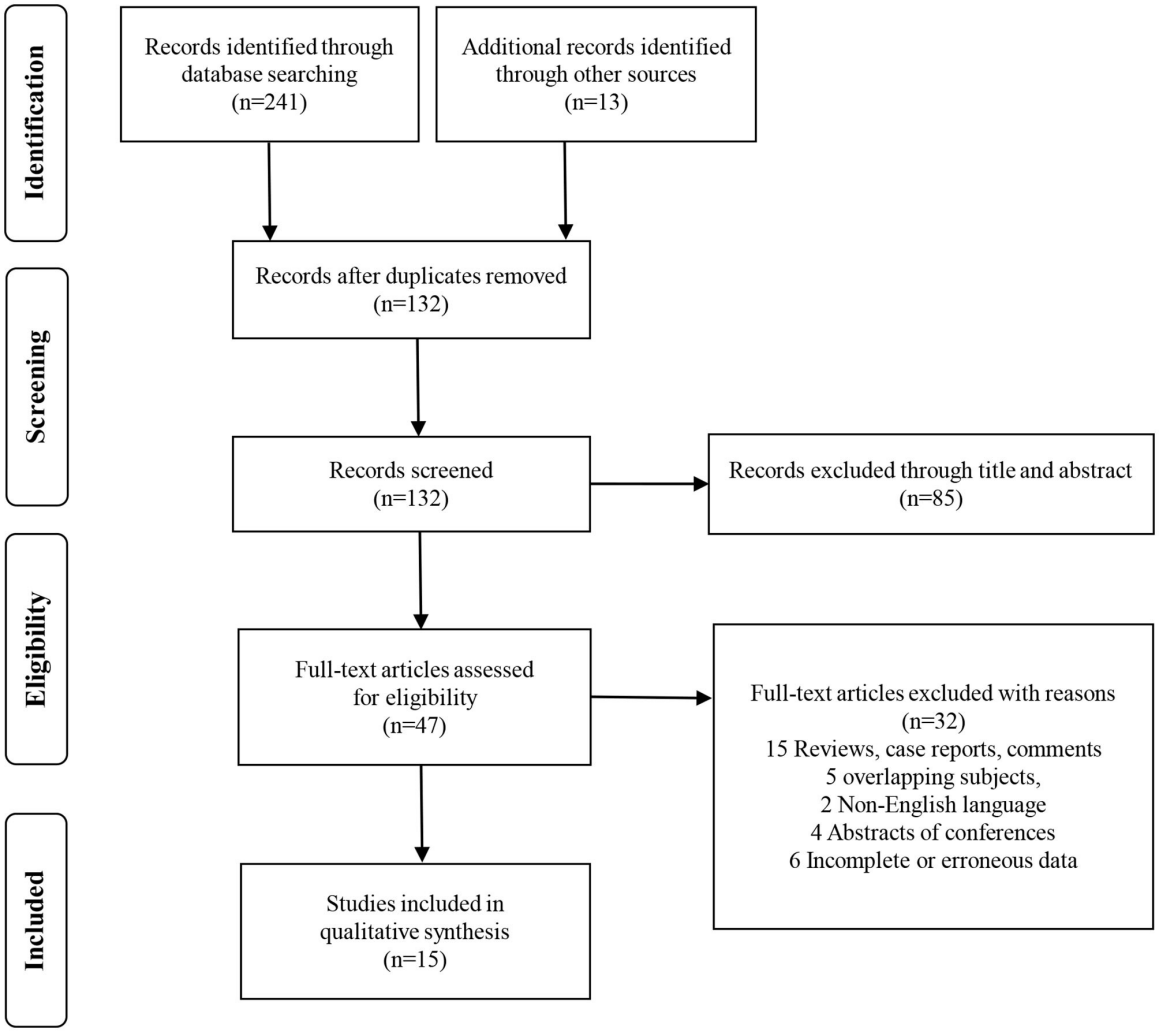
Flow diagram of literature search and selection process.

In total, 254 studies were identified by the initial screening, including 241 studies from database searches and 13 studies through other sources. After removing the duplicates, the remaining 132 records were excluded by scanning the title and the abstract. Forty-seven potential articles were screened carefully. Fifteen of these were ruled out for being reviews, case reports, and comments; five for having overlapping subjects; two for being in the non-English language; four for being abstracts of conferences; and six for lacking essential survival data. After scrupulous selection, 15 studies (21–35) were eventually enrolled in this meta-analysis. The selection process is shown in Figure 1. In total, 2,691 patients with RCC were included in this meta-analysis. The mean age of the patients ranged from 56 to 66 years old, the sample size ranged from 23 to 430 patients, the publication year ranged from 2007 to 2018, and the median follow-up time ranged from 10 to 108 months. Five studies were from Europe, three were from America, and seven were from Asia. Baseline characteristics of the eligible studies are shown in Table 1. NOS scores of all the included studies were above 6 (details are provided in Table 2). Eight studies were found on OS, six on CSS, two on PFS, and three on RFS.

**Table 1:** Baseline characteristics of eligible studies.

**References**	**Nation/region**	**Study type/date**	**Tumor entities**	**Stage**	**Treatment**	**No. of patients**	**Male/female**	**Mean age (range)/y**	**GPS/mGPS**	**Score**	**Outcome**	**Follow-up (range)/mo**
Ramsey et al. (21)	UK/Europe	Prospectively cohort study/2001–2005	mRCC	RCC^#^	α-interferon	119	85/35	60	GPS	0: 33 (27.7%) 1: 72 (60.5%) 2: 14 (11.8%)	CSS	10
Ramsey et al. (22)	UK/Europe	Prospectively cohort study/May 2005–Feb. 2006	mRCC	RCC^#^	α-interferon	23	18/5	60	GPS	0: 8 (34.8%) 1: 9 (39.1%) 2: 6 (26.1%)	CSS	23
Qayyum et al. (24)	UK/Europe	Prospectively cohort study/NA	ccRCC	ccRCC	RN	79	47/32	60 (39–82)	mGPS	0: 57 (72.2%) 1: 19 (24.0%) 2: 3 (3.8%)	CSS	93 (0.1–152)
Lamb et al. (23)	UK/Europe	Prospectively cohort study/Mar. 1997–Jul. 2007	ccRCC	ccRCC	RN	169	107/62	60	mGPS	0: 117 (69.2%) 1: 46 (27.2%) 2: 6 (3.6%)	OS, CSS	98
Tai et al. (25)	USA/North America	Prospectively cohort study/Nov. 2006–Jan. 2008	ccRCC	ccRCC	RN	129	83/46	62.0 (54.0–70.0)	mGPS	0: 80 (84.3%) 1: 27 (9.8%) 2: 22 (5.9%)	RFS	25.5 (12.0–32.4)
Lucca et al. (28)	Austria/Europe	Retrospective cohort study/2002–2014	ccRCC	ccRCC	RN/PN	430	257/173	65.5 (57–73)	GPS	0: 330 (76.8%) 1: 87 (20.2%) 2: 13 (3.0%)	RFS	40 (17–73)
Chen et al. (27)	China/Asia	Retrospective cohort study/2003–2012	ccRCC	ccRCC	RN/PN	406	253/153	58 (24–80)	mGPS/GPS	NA	OS	63 (1–151)
Baum et al. (26)	USA/North America	Prospectively cohort study/2005–2013	ccRCC	ccRCC	Nephrectomy	352	NA	58.8	mGPS	0: 267 (75.9%) 1: 38 (10.8%) 2: 47 (13.3%)	OS	31.6 (0.03–84)
Cho et al. (29)	South Korea/Asia	Prospectively cohort study/Jun. 1994–Jul. 2012	ccRCC	ccRCC	RN/PN	388	263/125	56.0 (18–81)	mGPS	0: 327 (84.3%) 1: 38 (9.8%) 2: 23 (5.9%)	RFS; CSS	53.7 (4–215)
Ishihara et al. (30)	Japan/Asia	Retrospective cohort study/2007–2014	mRCC	Mixed	sunitinib	71	50/21	64.0 (31–79)	mGPS	0: 53 (74.6%) 1: 10 (14.1%) 2: 8 (11.3%)	OS; PFS	17.0 (2.24–65.6)
Ohmura et al. (33)	Japan/Asia	Retrospective cohort study/Sept. 2009–Aug.2015	RCC (Mixed)	Mixed	Molecular-targeted agents	32	20/12	66 (33–82)	mGPS	0: 21 (65.6%) 1: 3 (9.4%) 2: 8 (25.0%)	OS; PFS	NA
Lorentz et al. (32)	USA/North America	Retrospective cohort study/2006–2016	ccRCC	ccRCC	RN	117	NA	NA	mGPS	0: 38 (32.3%) 1: 17 (14.4%) 2: 62 (53.3%)	OS	12.6 (4.8–32.4)
Harris et al. (31)	Japan/Asia	Retrospective cohort study/Jul. 2005–Jan. 2015	mRCC	Mixed	Molecular-targeted agents	181	144/37	63 (26–89)	mGPS	0: 92 (50.8%) 1: 36 (19.9%) 2: 53 (29.3%)	OS	NA
Owari et al. (35)	Japan/Asia	Retrospective cohort study/Jan. 2007–Dec. 2016	mRCC	RCC^#^	RT/Surgery	43	36/7	65.6 ± 12.1	GPS	0: 25 (58%) 1: 9 (21%) 2: 9 (21%)	CSS	108
Fukuda et al. (34)	Japan/Asia	Retrospective cohort study/Mar. 1986–Aug. 2015	mRCC	Mixed	CN	152	109/43	64.0 (61.5–64.8)	GPS	0: 47 (31%) 1: 59 (39%) 2: 46 (30%)	OS	100

NA, not available; HR, hazard ratio; CI, confidence interval; OS, overall survival; RFS, recurrence-free survival; PFS, progression-free survival; CSS, cancer-specific survival; RCC, renal cell cancer; ccRCC, clear cell renal cell carcinoma; GPS, Glasgow prognostic score; mGPS, modified Glasgow prognostic score; RN, radical nephrectomy; PN, partial nephrectomy; CN, cytoreductive nephrectomy; RT, radiotherapy.

# *represents specific tumor entity was not available*.

**Table 2:** Newcastle–Ottawa scale (NOS) for quality assessment of cohort studies.

**References**	**Selection**				**Comparability**		**Outcomes**			**Scores**
	**Representativeness** **of the exposed cohort**	**Selection of the** **non-exposed cohort**	**Ascertainment** **of exposure**	**Outcome of** **interest was not present at the start of study**	**Comparability** **of cohorts on** **the basis**	**Design analysis**	**Assessment** **of outcome**	**Follow-up** **long enough** **for outcomes**	**Adequacy** **of follow-up**	
Ramsey et al. (21)	⋆	⋆	⋆	⋆	⋆	⋆	–	⋆	⋆	8
Ramsey et al. (22)	⋆	⋆	–	–	⋆	⋆	–	⋆	⋆	6
Qayyum et al. (24)	–	–	⋆	⋆	⋆	⋆	–	⋆	⋆	6
Lamb et al. (23)	⋆	–	⋆	⋆	⋆	⋆	⋆	⋆	⋆	8
Tai et al. (25)	⋆	⋆	⋆	⋆	–	⋆	–	⋆	⋆	7
Lucca et al. (28)	⋆	–	⋆	⋆	⋆	⋆	⋆	–	⋆	7
Chen et al. (27)	⋆	–	⋆	–	⋆	⋆	–	⋆	⋆	6
Baum et al. (26)	⋆	⋆	⋆	⋆	⋆		–	⋆	–	6
Cho et al. (29)	–	–	⋆	⋆	⋆	⋆	⋆	⋆	⋆	7
Ishihara et al. (30)	⋆	⋆	⋆	–	⋆	⋆	⋆	–	⋆	7
Ohmura et al. (33)	⋆	⋆	⋆	⋆	⋆	⋆	⋆	–	–	7
Lorentz et al. (32)	⋆	⋆	⋆	–	–	⋆	–	⋆	⋆	6
Harris et al. (31)	–	–	⋆	⋆	⋆	⋆	–	⋆	⋆	6
Owari et al. (35)	⋆	⋆	⋆	–	⋆	⋆	–	–	⋆	6
Fukuda et al. (34)	–	–	⋆	⋆	⋆	⋆	⋆	–	⋆	6

A maximum of one star for each item within the selection and outcome categories.

A maximum of two stars could be given for the comparability category.

Up to nine stars could be awarded.

*Each star (⋆) represents one score*.

### Prognostic Value of GPS/mGPS for OS in RCC

**Figure 2:**
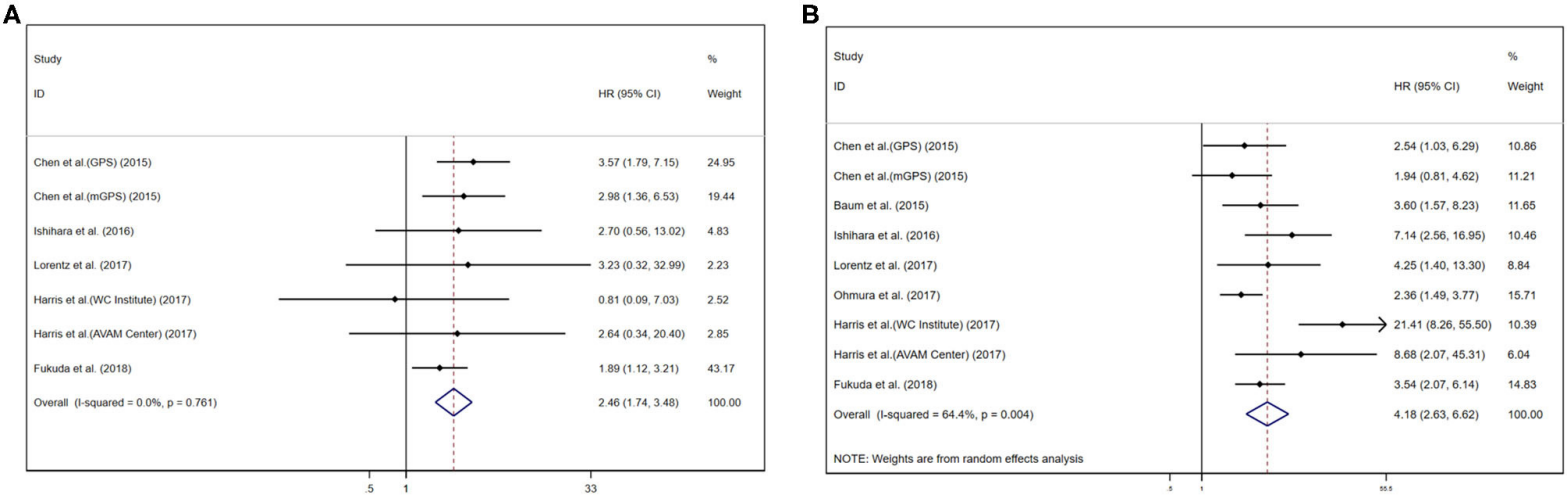
Forest plots of pooled GPS/mGPS for OS in RCC. **(A)** GPS/mGPS of 1, **(B)** GPS/mGPS of 2. HR, hazard ratio; CI, confidence interval; GPS, Glasgow prognostic score; mGPS, modified Glasgow prognostic score; OS, overall survival; RCC, renal cell carcinoma; WC institute, Winship Cancer Institute; AVAM cancer, Atlanta Veterans Administration Medical Center.

Pooled results were showed in the forest plots (Figures 2A,B). Eleven studies showed that higher GPS/mGPS (GPS/mGPS of 2) had a significant association with poorer OS in patients with RCC [HR 4.18, 95% CI (2.63, 6.62), *P* < 0.001] with significant heterogeneity (*I*^2^ = 64.4%, *P*_heterogeneity_ = 0.004). The fixed effects model was used to analyze the relationship between them. In addition, seven studies showed that GPS/mGPS of 1 had a significant association with inferior OS in patients with RCC [HR 2.46, 95% CI (1.74, 3.48), *P* < 0.001] with slight heterogeneity (*I*^2^ = 0%, *P*_heterogeneity_ = 0.761).

Subgroup analyses were performed for OS based on stage, their inherent difference, and regions. For patients with RCC, the pooled results revealed that GPS/mGPS of 2 predicted inferior OS in patients with metastatic tumors [HR 5.74, 95% CI (3.91, 8.42), *P* < 0.001], mGPS [HR 3.75, 95% CI (2.77, 5.09), *P* < 0.001], and patients in European and American countries [HR 3.78, 95% CI (2.63, 5.43), *P* < 0.001]. Similarly, the results revealed that GPS/mGPS of 1 predicted inferior OS in patients with non-metastatic tumors [HR 3.30, 95% CI (1.96, 5.54), *P* < 0.001], mGPS [HR 2.64, 95% CI (1.43, 4.86), *P* < 0.001], and patients in Asian countries [HR 2.53, 95% CI (1.77, 3.61), *P* < 0.001]. The pooled results were shown in Table 3 and Supplementary Figure 1.

**Table 3:** Summary of subgroup analysis results of GPS/mGPS in OS.

**Subgroup**	**GPS/mGPS score of 1**					**GPS/mGPS score of 2**				
	**Studies, no**.	**HR (95% Cl)**	***P*-value**	***I*^**2**^ (%)**	***P*_**heterogeneity**_**	**Studies, no**.	**HR (95% Cl)**	***P*-value**	***I*^**2**^ (%)**	***P*_**heterogeneity**_**
Overall	7	2.46 (1.74–3.48)	<0.001	0	0.761	9	4.18 (2.63–6.62)	<0.001	64.4	0.004
**Stage**										
Metastatic	5	1.95 (1.23–3.10)	0.005	0	0.899	5	5.74 (3.91–8.42)	<0.001	77.0	0.025
Non-metastatic	2	3.30 (1.96–5.54)	<0.001	0	0.735	4	2.49 (1.77–3.49)	<0.001	0	0.77
**GPS or mGPS**										
GPS	2	2.39 (1.57–3.63)	<0.001	51.3	0.152	2	3.24 (2.03–5.17)	<0.001	0	0.538
mGPS	5	2.64 (1.43–4.86)	0.002	0	0.870	7	3.75 (2.77–5.09)	<0.001	72.5	0.001
**Region**										
Asian countries	5	2.53 (1.77–3.61)	<0.001	0	0.673	5	3.42 (2.39–4.89)	<0.001	11.8	0.339
European and American countries	2	1.52 (0.34–6.75)	0.584	0	0.439	4	3.78 (2.63–5.43)	<0.001	83.2	<0.001

*HR, hazard ratio; CI, confidence interval; OS, overall survival; GPS, Glasgow prognostic score; mGPS, modified Glasgow prognostic score*.

### Prognostic Value of GPS/mGPS for CSS in RCC

**Figure 3:**
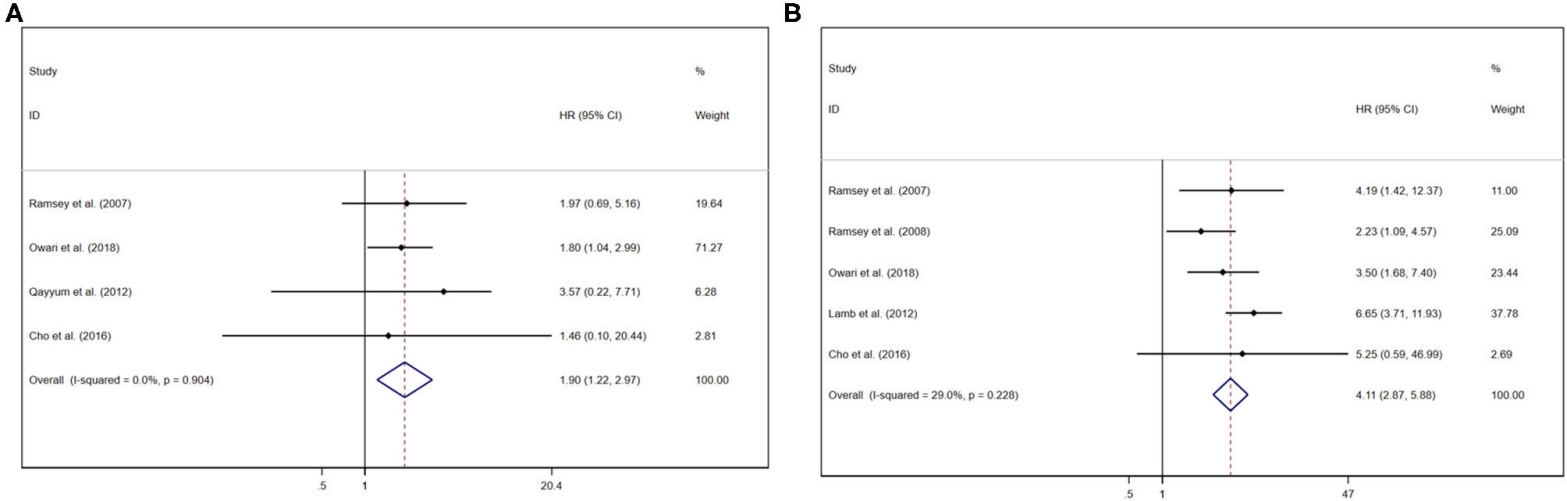
Forest plots of pooled GPS/mGPS for CSS in RCC. **(A)** GPS/mGPS of 1, **(B)** GPS/mGPS of 2. HR, hazard ratio; CI, confidence interval; GPS, Glasgow prognostic score; mGPS, modified Glasgow prognostic score; CSS, cancer specific survival; RCC, renal cell carcinoma.

Pooled results were showed in the forest plots (Figures 3A,B). Five studies showed that GPS/mGPS of 2 had a significant association with inferior CSS in patients with RCC [HR 4.11, 95% CI (2.87, 5.88), *P* < 0.001] with slight heterogeneity (*I*^2^ = 0%, *P*_heterogeneity_ = 0.228). In addition, four studies showed that GPS/mGPS of 1 had a significant association with inferior CSS in patients with RCC [HR 1.90, 95% CI (1.22, 2.97), *P* = 0.005] with slight heterogeneity (*I*^2^ = 0%, *P*_heterogeneity_ = 0.904). The fixed effects model was used to analyze both of these relationships.

### Prognostic Value of GPS/mGPS for RFS in RCC

**Figure 4:**
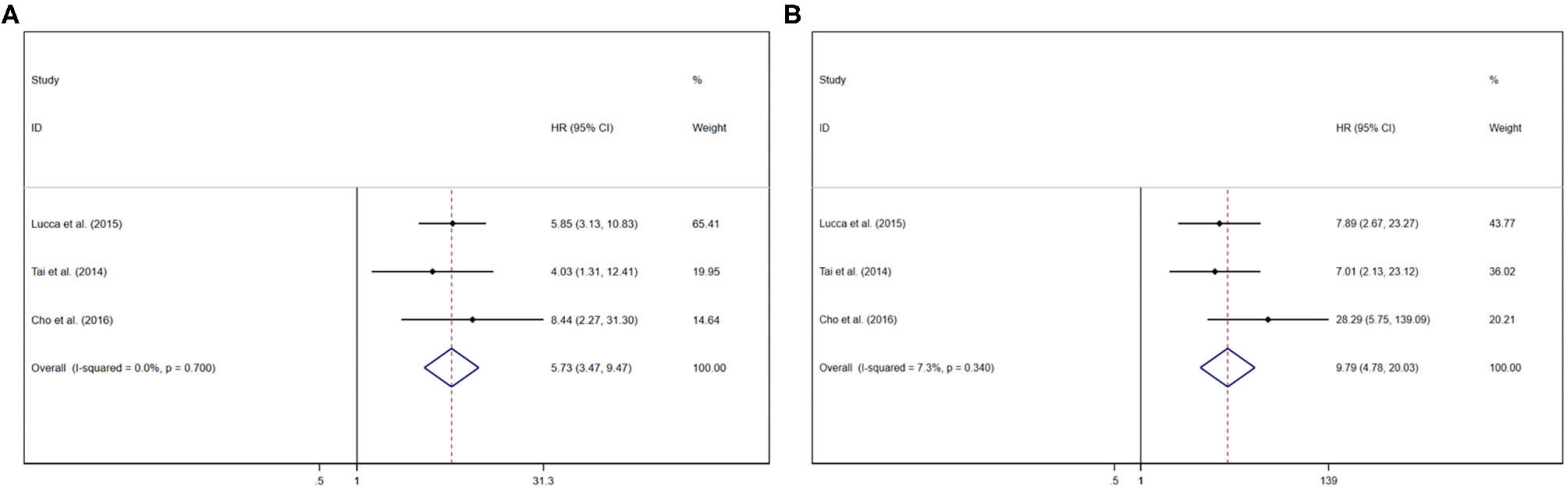
Forest plots of pooled GPS/mGPS for RFS in RCC. **(A)** GPS/mGPS of 1, **(B)** GPS/mGPS of 2. HR, hazard ratio; CI, confidence interval; GPS, Glasgow prognostic score; mGPS, modified Glasgow prognostic score; RFS, recurrence-free survival; RCC, renal cell carcinoma.

Pooled results were showed in the forest plots (Figures 4A,B). Three studies showed that GPS/mGPS of 2 had a significant association with inferior RFS in patients with RCC [HR 9.79, 95% CI (4.78, 20.03), *P* < 0.001] with slight heterogeneity (*I*^2^ = 7.30%, *P*_heterogeneity_ = 0.34). The fixed effects model was used to analyze both these relationships. Similarly, three studies showed that GPS/mGPS of 1 had a significant association with inferior RFS in patients with RCC [HR 5.73, 95% CI (3.47, 9.47), *P* < 0.001] with slight heterogeneity (*I*^2^ = 0%, *P*_heterogeneity_ = 0.70).

### Prognostic Value of GPS/mGPS for PFS in RCC

**Figure 5:**
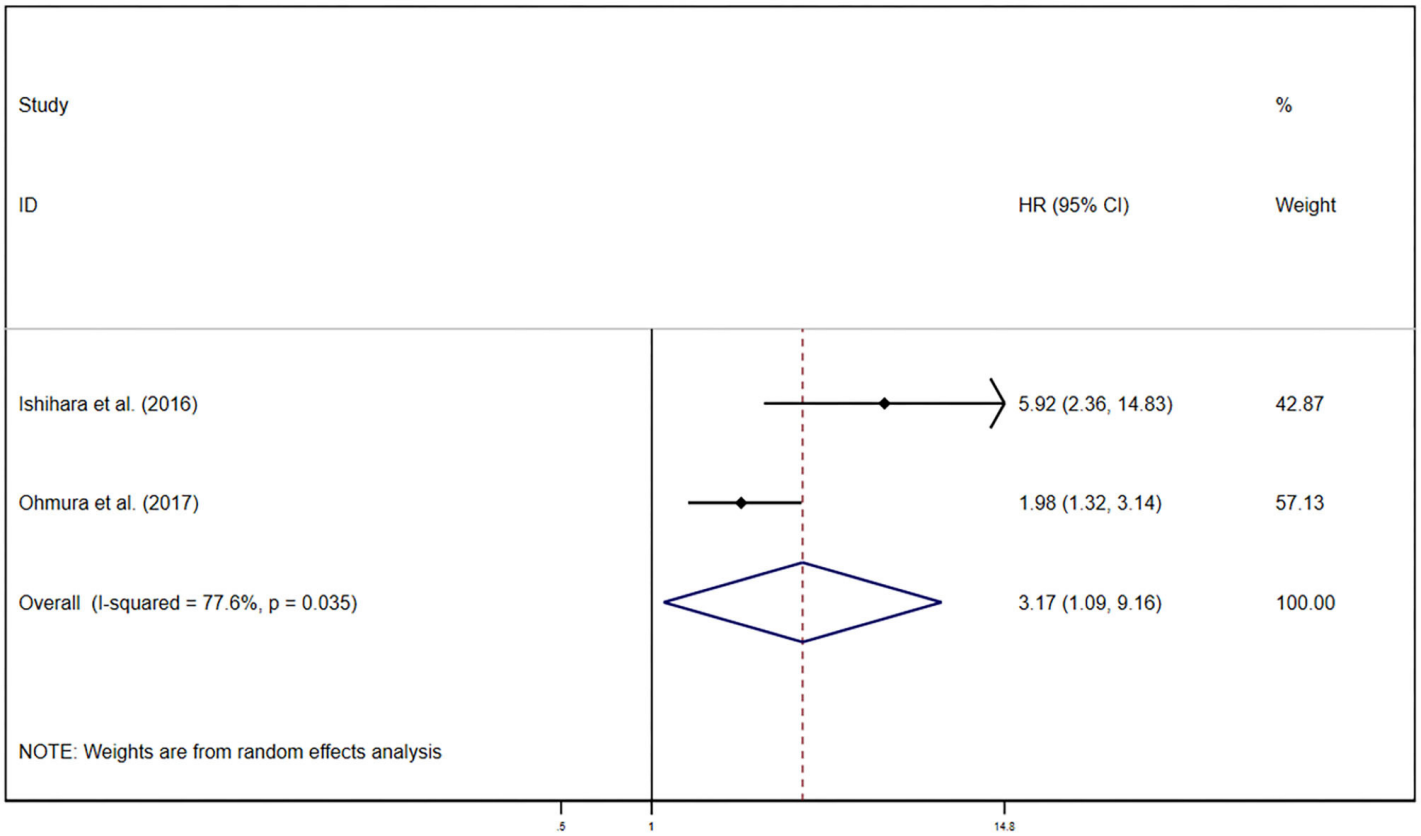
Forest plots of pooled GPS/mGPS of 2 for PFS in RCC. HR, hazard ratio; CI, confidence interval; GPS, Glasgow prognostic score; mGPS, modified Glasgow prognostic score; PFS, progression-free survival; RCC, renal cell carcinoma; RCC, renal cell carcinoma.

Pooled results were showed in the forest plots (Figure 5). Only two studies illustrated that GPS/mGPS of 2 had an association with inferior PFS in patients with RCC [HR 3.17, 95% CI (1.09, 9.16), *P* = 0.03] with heterogeneity (*I*^2^ = 77.6%, *P*_heterogeneity_ = 0.04). The randomized effects model was used to analyze this relationship.

### Sensitivity Analysis and Publication Bias

A sensitivity analysis was performed by excluding any single study in sequence at a time to assess how this affected the pooled results. The results indicated that the pooled HRs for OS, CSS, and RFS did not change significantly, suggesting the stability of the results. Publication bias was evaluated by Egger's test. A study was considered to have a significant publication bias when *P* < 0.05. The results of Egger's test indicated that there was no evidence of publication bias in OS (*P* = 0.967), CSS (*P* = 0.967), and RFS (*P* = 0.967) with GPS/mGPS of 1, whereas OS (*P* = 0.144), CSS (*P* = 0.848), RFS (*P* = 0.228) with GPS/mGPS of 2 (details are provided in Supplementary Figures 2–4).

## Discussion

The results of the present study showed that higher GPS/mGPS was significantly associated with poorer OS, CSS, RFS, and PFS, especially when GPS/mGPS was equal to 2. GPS and mGPS could be recognized as significant prognostic biomarkers for predicting survival outcomes in patients with RCC. However, there was a significant heterogeneity among patients with GPS/mGPS of 2. In addition, subgroup analyses were performed based on stage, regions, and their inherent difference. According to subgroup analyses, patients with metastatic RCC had inferior OS than those with non-metastatic RCC when GPS/mGPS was 2, whereas pooled HR showed contrary results when GPS/mGPS was 1 because only a single cohort was analyzed and there was a lack of representativeness. mGPS showed an inferior prognosis than GPS, which indicated that mGPS appeared to be a better specific factor. Moreover, it was more specific for predicting an inferior prognosis in European and American patients with a higher grade of GPS/mGPSthan in Asian patients, whereas the contrary was observed when GPS/mGPS was equal to 1. Subgroup analysis revealed a low impact on RFS and PFS because of inadequate studies; therefore, further evaluations are needed. Caution is necessary when generalizing these results, given the between-cohort heterogeneity.

Changes in the quantity and levels of white blood cells, platelets, lymphocytes, neutrophils, CRP, and albumin have been found to play a dominant role in the inflammatory response, triggered by harmful stimulation and conditions, such as infection, tissue injury, and tissue malfunction (36). CRP is a typical acute protein produced by hepatocytes and induced by cytokines, especially IL-6 (37), whereas albumin is produced only in the liver and can reflect the nutritional status of patients. Furthermore, undernutrition is associated with a poor prognosis (38). Since the initial studies used the systemic inflammatory response, an independent prognostic value has been reported for operable colorectal cancer (39), as well as gastrointestinal cancer (9), non-small cell lung cancer (10), colorectal neoplasms (11), urothelial carcinoma (12), and RCC (13). An early study (40) reported that albumin levels decreased as circulating CRP levels increased, and this relationship was observed in multiple types of tumors. In particular, interestingly, CRP levels elevated to more than 10 mg/l, and albumin levels reduced to <32 g/l showed the highest HRs on OS (41).

GPS/mGPS, which assesses the grade of systemic inflammatory response, is completely based on objective criteria and is convenient to measure, is routinely available, and is well-normalized worldwide. Considering the characteristic of ordinal categorical variables, which had been ignored in previous systematic reviews and meta-analyses, we considered that GPS/mGPS should be hierarchic. Thus, GPS/mGPS of 0 was regarded as low risk, GPS/mGPS of 1 was regarded as medium risk, and GPS/mGPS of 2 was regarded as high risk. In addition, we calculated HRs separately when GPS/mGPS was equal to 1 and 2, referring to GPS/mGPS of 0 for distinguishing between the different grades, which made our study more convincing. This is extremely important, especially in patients with advanced cancer. It has been confirmed that systemic inflammation is associated with progressive nutritional and functional decline and the subsequent poor outcomes as well as quality of life parameters in patients with advanced cancer (42, 43). The systemic inflammatory response in these patients has been recognized as a chronic inflammatory cascade, resulting in profound alterations at the genomic, intracellular, cellular, and systemic levels (44, 45). In a previous study, genomic changes contributed to the chronic activation of the JAK/STAT pathway in tumor cells, and under this condition, IL-6 production was out of control, leading to unregulated inflammatory cascade at cellular and systemic levels via increased CRP (46). Recently, accumulative evidences have proved that immune checkpoint inhibitor conducts durable and effective response to block programmed cell death 1 receptor (PD-1) and PD ligand 1 (PD-L1) by reducing tumor volume and develops the survival outcome of patients with several cancers such as renal cell carcinoma. The application of inhibitors of PD-1 and its ligand PD-L1 was considered as the landscape of the therapy in advanced RCC (47). Cancer cell can highly express PD-L1 to avoid immune monitoring and escape immune system that leads to poor prognosis. Therefore, PD-L1 expression in RCC may also serve as a biomarker as well as GPS/mGPS. However, both of these biomarkers do not have a direct evidence to prove that PD-1/PD-L1 and GPS/mGPS have a great correlation.

This study has several limitations. First, studies that investigated the correlation between PFS/RFS and GPS/mGPS were scarce; therefore, we could not obtain robust conclusions via these endpoint analyses. Second, there was a lack of research from South America, Africa, and Oceania, leading to inadequate included studies. Third, retrieving eligible studies published only in English may have neglected studies published in other languages. Fourth, a part of survival information was unavailable; therefore, we used Engauge Digitizer to digitize and extract Kaplan–Meier curve-related survival information, which generated inevitable bias. Fifth, relevant studies were insufficient in some subgroup analyses, which resulted in an uncontrollable bias, such as the subgroup on stage and region. Sixth, the CRP and albumin levels were obtained from peripheral blood before the operation or treatment and were thus easily susceptible by patients' elementary conditions such as age, tumor burden, histological features, disease stage, infection, inflammatory disease, chronic disease, and specific medications and individual factors such as smoking and drinking. Seventh, this study was not able to conduct a subgroup analysis with regard to the different individual tumor entities of the renal cell carcinoma because the data were insufficient and scattered. Eighth, only cohort studies were included in this study; therefore, the results of the present meta-analysis should be cautiously interpreted.

## Conclusion

In summary, this meta-analysis demonstrated that GPS and mGPS are effective biomarkers for predicting prognosis in patients with RCC, and higher GPS and mGPS are closely related to inferior survival outcomes. More randomized controlled trials are necessary to investigate the promising value of hematological parameters in the future.

## Data Availability Statement

All datasets generated for this study are included in the article/Supplementary Material.

## Author Contributions

TT and YG: conceptualization. TT, YG, and HX: methodology. TT: writing, editing, and revision. LW and JP: supervision and review. All authors: contributed to data analysis, drafting or revising the article, gave final approval of the version to be published, and agreed to be accountable for all aspects of work. All authors contributed to the article and approved the submitted version.

## Conflict of Interest

The authors declare that the research was conducted in the absence of any commercial or financial relationships that could be construed as a potential conflict of interest.

## References

[1] Siegel RL Miller KD Jemal A Cancer statistics, 2020. CA Cancer J Clin. (2020) 70:7–30. 10.3322/caac.21590 31912902

[2] Cheng G Liu D Liang H Yang H Chen K Zhang X. A cluster of long non-coding RNAs exhibit diagnostic and prognostic values in renal cell carcinoma. Aging. (2019) 11:9597–615. 10.18632/aging.102407 31727869 PMC6874440

[3] Sun M Choueiri TK Kidney cancer: recurrence in renal cell carcinoma: the work is not done. Nat Rev Urol. (2016) 13:246–7. 10.1038/nrurol.2016.57 27030528

[4] Siegel R Naishadham D Jemal A. Cancer statistics, 2013. CA A Cancer J Clin. (2013) 63:11–30. 10.3322/caac.21166 23335087

[5] Kroeger N Zimmermann U Burchardt M Pantuck AJ. Prognostication in localised renal cell carcinoma. Lancet Oncol. (2015) 16:603–4. 10.1016/S1470-2045(15)70227-5 25979596

[6] Song J Song F Liu K Zhang W Luo R Tang Y. Multi-omics analysis reveals epithelial-mesenchymal transition-related gene FOXM1 as a novel prognostic biomarker in clear cell renal carcinoma. Aging. (2019) 11:10316–37. 10.18632/aging.102459 31743108 PMC6914426

[7] Forrest LM McMillan DC McArdle CS Angerson WJ Dunlop DJ. Evaluation of cumulative prognostic scores based on the systemic inflammatory response in patients with inoperable non-small-cell lung cancer. Br J Cancer. (2003) 89:1028–30. 10.1038/sj.bjc.6601242 12966420 PMC2376960

[8] Forrest LM McMillan DC McArdle CS Angerson WJ Dunlop DJ. Comparison of an inflammation-based prognostic score (GPS) with performance status (ECOG) in patients receiving platinum-based chemotherapy for inoperable non-small-cell lung cancer. Br J Cancer. (2004) 90:1704–6. 10.1038/sj.bjc.6601789 15150622 PMC2409737

[9] McMillan DC Crozier JEM Canna K Angerson WJ McArdle CS. Evaluation of an inflammation-based prognostic score (GPS) in patients undergoing resection for colon and rectal cancer. Int J Colorectal Dis. (2007) 22:881–6. 10.1007/s00384-006-0259-6 17245566

[10] Hsueh S-W Liu K-H Hung C-Y Kuo Y-C Tsai C-Y Hsu J-T. Significance of the glasgow prognostic score in predicting the postoperative outcome of patients with stage III gastric cancer. J Clin Med. (2019) 8:1448. 10.3390/jcm8091448 31547247 PMC6780196

[11] Lindenmann J Fink-Neuboeck N Taucher V Pichler M Posch F Brcic L. Prediction of postoperative clinical outcomes in resected stage I non-small cell lung cancer focusing on the preoperative glasgow prognostic score. Cancers. (2020) 12:152. 10.3390/cancers12010152 31936329 PMC7016624

[12] Tsuchihashi K Ito M Moriwaki T Fukuoka S Taniguchi H Takashima A. Role of predictive value of the modified glasgow prognostic score for later-line chemotherapy in patients with metastatic colorectal cancer. Clin Colorectal Cancer. (2018) 17:e687–97. 10.1016/j.clcc.2018.07.004 30149986

[13] Kimura S D'Andrea D Soria F Foerster B Abufaraj M Vartolomei MD. Prognostic value of modified Glasgow Prognostic Score in non-muscle-invasive bladder cancer. Urol Oncol. (2019) 37:179. e119–79.e128. 10.1016/j.urolonc.2018.11.005 30580906

[14] Tsujino T Komura K Matsunaga T Yoshikawa Y Takai T Uchimoto T. Preoperative measurement of the modified glasgow prognostic score predicts patient survival in non-metastatic renal cell carcinoma prior to nephrectomy. Ann Surg Oncol. (2017) 24:2787–93. 10.1245/s10434-017-5948-6 28643013

[15] Shim SR Kim SJ Kim SI Cho DS. Prognostic value of the glasgow prognostic score in renal cell carcinoma: a meta-analysis. World J Urol. (2017) 35:771–80. 10.1007/s00345-016-1940-1 27665441

[16] Hu X Wang Y Yang WX Dou WC Shao YX Li X. Modified Glasgow prognostic score as a prognostic factor for renal cell carcinomas: a systematic review and meta-analysis. Cancer Manag Res. (2019) 11:6163–73. 10.2147/CMAR.S208839 31308752 PMC6613602

[17] Qi F Xu Y Zheng Y Li X Gao Y. Pre-treatment Glasgow prognostic score and modified Glasgow prognostic score may be potential prognostic biomarkers in urological cancers: a systematic review and meta-analysis. Ann Transl Med. (2019) 7:531. 10.21037/atm.2019.09.160 31807513 PMC6861778

[18] Hua X Chen J Su Y Liang C. Identification of an immune-related risk signature for predicting prognosis in clear cell renal cell carcinoma. Aging. (2020) 12:2302–32. 10.18632/aging.102746 32028264 PMC7041771

[19] Xie L Li H Zhang L Ma X Dang Y Guo J. Autophagy-related gene: a novel diagnosis and prognosis marker for kidney renal clear cell carcinoma. Aging. (2020) 12:1828–42. 10.18632/aging.102715 32003756 PMC7053637

[20] Yang W Zhang K Li L Ma K Hong B Gong Y. Discovery and validation of the prognostic value of the lncRNAs encoding snoRNAs in patients with clear cell renal cell carcinoma. Aging. (2020) 12:4424–44. 10.18632/aging.102894 32126023 PMC7093172

[21] Ramsey S Lamb GW Aitchison M Graham J McMillan DC. Evaluation of an inflammation-based prognostic score in patients with metastatic renal cancer. Cancer. (2007) 109:205–12. 10.1002/cncr.22400 17149754

[22] Ramsey S Aitchison M Graham J McMillan DC The longitudinal relationship between the systemic inflammatory response, circulating T-lymphocytes, interleukin-6 and−10 in patients undergoing immunotherapy for metastatic renal cancer. BJU Int. (2008) 102:125–9. 10.1111/j.1464-410X.2008.07466.x 18336617

[23] Lamb GW Aitchison M Ramsey S Housley SL McMillan DC. Clinical utility of the Glasgow prognostic score in patients undergoing curative nephrectomy for renal clear cell cancer: basis of new prognostic scoring systems. Br J Cancer. (2012) 106:279–83. 10.1038/bjc.2011.556 22166802 PMC3261680

[24] Qayyum T McArdle PA Lamb GW Going JJ Orange C Seywright M. Prospective study of the role of inflammation in renal cancer. Urol Int. (2012) 88:277–81. 10.1159/000334971 22377628

[25] Tai CG Johnson TV Abbasi A Herrell L Harris WB Kucuk O. External validation of the modified Glasgow prognostic score for renal cancer. Indian J Urol. (2014) 30:33–7. 10.4103/0970-1591.124203 24497679 PMC3897050

[26] Baum Y De la Calle C Patil D Bausum A Huang J Alemozaffar M Mp35-14 commonly-used cost-effective pre-operative inflammatory markers provide prognostic information in patients with localized clear cell renal cell carcinoma. J Urol. (2015) 193:e424 10.1016/j.juro.2015.02.1115

[27] Chen Z Shao Y Fan M Zhuang Q Wang K Cao W. Prognostic significance of preoperative C-reactive protein: albumin ratio in patients with clear cell renal cell carcinoma. Int J Clin Exp Pathol. (2015) 8:14893–900. 26823819 PMC4713605

[28] Lucca I de Martino M Hofbauer SL Zamani N Shariat SF Klatte T. Comparison of the prognostic value of pretreatment measurements of systemic inflammatory response in patients undergoing curative resection of clear cell renal cell carcinoma. World J Urol. (2015) 33:2045–52. 10.1007/s00345-015-1559-7 25894368

[29] Cho DS Kim SI Choo SH Jang SH Ahn HS Kim SJ. Prognostic significance of modified Glasgow prognostic score in patients with non-metastatic clear cell renal cell carcinoma. Scand J Urol. (2016) 50:186–91. 10.3109/21681805.2015.1136677 26878156

[30] Ishihara H Kondo T Omae K Takagi T Iizuka J Kobayashi H. Sarcopenia and the modified Glasgow prognostic score are significant predictors of survival among patients with metastatic renal cell carcinoma who are receiving first-line sunitinib treatment. Target Oncol. (2016) 11:605–17. 10.1007/s11523-016-0430-0 27023922

[31] Harris WB Zhang C Liu Y Robertson DK Akbashev MY Lingerfelt BM. Time-dependent effects of prognostic biomarkers of systemic inflammation in patients with metastatic renal cell carcinoma. Tumour Biol. (2017) 39:1010428317705514. 10.1177/1010428317705514 28618965

[32] Lorentz A Gupta M Broggi M Leung A Patil D Master V Mp16-03 utility of inflammatory markers in prognosis for patients with renal cell carcinoma and vena cava tumor thrombus. J Urol. (2017) 197:e180 10.1016/j.juro.2017.02.509

[33] Ohmura H Uchino K Kajitani T Sakamoto N Baba E. Predictive value of the modified Glasgow prognostic score for the therapeutic effects of molecular-targeted drugs on advanced renal cell carcinoma. Mol Clin Oncol. (2017) 6:669–75. 10.3892/mco.2017.1205 28515920 PMC5431320

[34] Fukuda H Takagi T Kondo T Shimizu S Tanabe K. Predictive value of inflammation-based prognostic scores in patients with metastatic renal cell carcinoma treated with cytoreductive nephrectomy. Oncotarget. (2018) 9:14296–305. 10.18632/oncotarget.24507 29581844 PMC5865670

[35] Owari T Miyake M Nakai Y Morizawa Y Hori S Anai S. A genitourinary cancer-specific scoring system for the prediction of survival in patients with bone metastasis: a retrospective analysis of prostate cancer, renal cell carcinoma, and urothelial carcinoma. Anticancer Res. (2018) 38:3097–103. 10.21873/anticanres.12568 29715146

[36] Medzhitov R. Origin and physiological roles of inflammation. Nature. (2008) 454:428–35. 10.1038/nature07201 18650913

[37] Pepys MB Hirschfield GM. C-reactive protein: a critical update. J Clin Investig. (2003) 111:1805–12. 10.1172/JCI200318921 12813013 PMC161431

[38] Ballmer PE. Causes and mechanisms of hypoalbuminaemia. Clin Nutr. (2001) 20:271–3. 10.1054/clnu.2001.0439 11407876

[39] McMillan DC Wotherspoon HA Fearon KC Sturgeon C Cooke TG McArdle CS. A prospective study of tumor recurrence and the acute-phase response after apparently curative colorectal cancer surgery. Am J Surg. (1995) 170:319–22. 10.1016/S0002-9610(99)80296-7 7573721

[40] McMillan DC Elahi MM Sattar N Angerson WJ Johnstone J McArdle CS. Measurement of the systemic inflammatory response predicts cancer-specific and non-cancer survival in patients with cancer. Nutr Cancer. (2001) 41:64–9. 10.1080/01635581.2001.9680613 12094630

[41] Howren MB Lamkin DM Suls J. Associations of depression with C-reactive protein, IL-1, and IL-6: a meta-analysis. Psychosomatic Med. (2009) 71:171–86. 10.1097/PSY.0b013e3181907c1b 19188531

[42] McMillan DC Systemic inflammation, nutritional status and survival in patients with cancer. Curr Opin Clin Nutr Metabolic Care. (2009) 12:223–6. 10.1097/MCO.0b013e32832a7902 19318937

[43] Laird BJA Fallon M Hjermstad MJ Tuck S Kaasa S Klepstad P. Quality of life in patients with advanced cancer: differential association with performance status and systemic inflammatory response. J Clin Oncol. (2016) 34:2769–75. 10.1200/JCO.2015.65.7742 27354484 PMC5019748

[44] Diakos CI Charles KA McMillan DC Clarke SJ. Cancer-related inflammation and treatment effectiveness. Lancet Oncol. (2014) 15:e493–503. 10.1016/S1470-2045(14)70263-3 25281468

[45] Sreeramkumar V Adrover JM Ballesteros I Cuartero MI Rossaint J Bilbao I. Neutrophils scan for activated platelets to initiate inflammation. Science. (2014) 346:1234–8. 10.1126/science.1256478 25477463 PMC4280847

[46] Dolan RD McSorley ST Horgan PG Laird B McMillan DC. The role of the systemic inflammatory response in predicting outcomes in patients with advanced inoperable cancer: systematic review and meta-analysis. Crit Rev Oncol Hematol. (2017) 116:134–46. 10.1016/j.critrevonc.2017.06.002 28693795

[47] Xu W Atkins MB McDermott DF. Checkpoint inhibitor immunotherapy in kidney cancer. Nat Rev Urol. (2020) 17:137–50. 10.1038/s41585-020-0282-3 32020040

