# Supplementary material for: A Meta-Analysis of Glasgow Prognostic Score and Modified Glasgow Prognostic Score as Biomarkers for Predicting Survival Outcome in Renal Cell Carcinoma
Source: Front Oncol. 2020 Sep 17;10:1541. doi: 10.3389/fonc.2020.01541 (PMC7527435; doi:10.3389/fonc.2020.01541)
Supplement: Supplementary file 1 [file Table_1.DOCX]

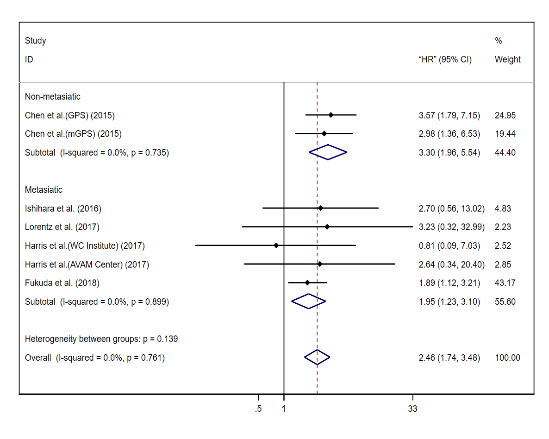

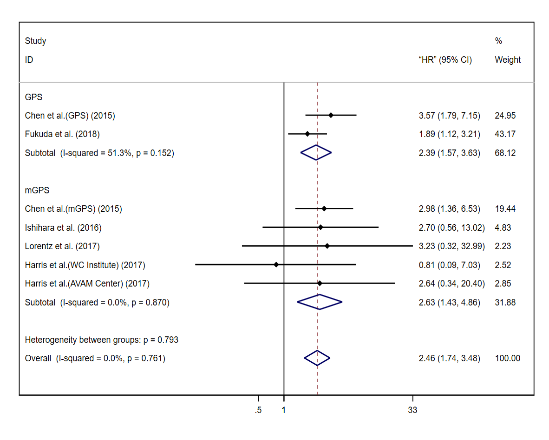

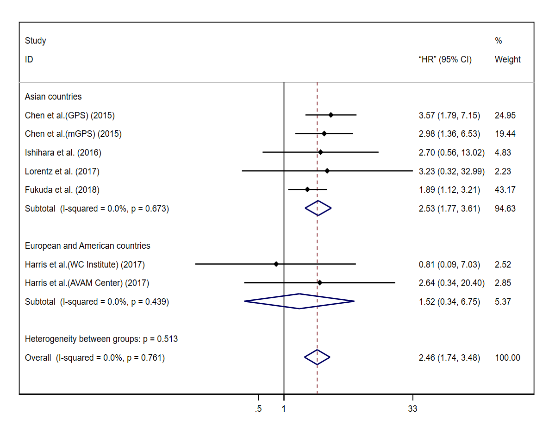


**A**

**B**

**C**


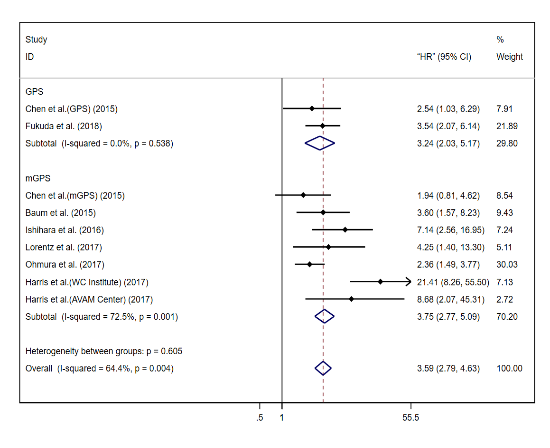

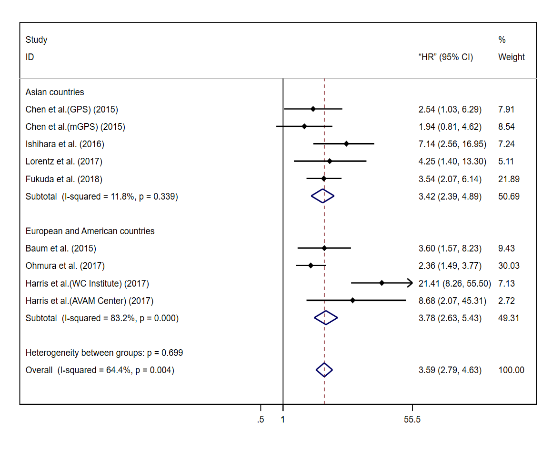


**E**

**F**


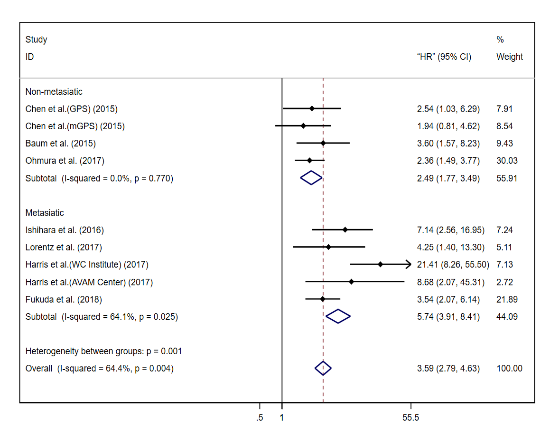


**D**

Figure S1. Forest plots of subgroup analysis of pooled GPS/mGPS for OS in RCC. GPS/mGPS of 1: a stage, b GPS or mGPS, c region; GPS/mGPS of 2: d stage, e GPS or mGPS, f region

Abbreviations: HR, hazard ratio; CI, confidence interval; GPS, Glasgow prognostic score; mGPS, modified Glasgow prognostic score; OS, overall survival; RCC, renal cell carcinoma; RCC, renal cell carcinoma; WC institute, Winship Cancer Institute; AVAM cancer, Atlanta Veterans Administration Medical Center

**B**

**A**

**D**

**C**

Figure S2. Egger's test, and sensitivity analysis and of pooled GPS/mGPS for OS in RCC. a, b GPS/mGPS of 1; c, d GPS/mGPS of 2

Abbreviations: CI, confidence interval; GPS, Glasgow prognostic score; mGPS, modified Glasgow prognostic score; OS, overall survival; RCC, renal cell carcinoma; WC institute, Winship Cancer Institute; AVAM cancer, Atlanta Veterans Administration Medical Center

**A**

**B**

**C**

**D**

Figure S3. Egger's test and sensitivity analysis and of pooled GPS/mGPS on CSS in patients with RCC. a, b GPS/mGPS of 1; c, d GPS/mGPS of 2

Abbreviations: CI, confidence interval; GPS, Glasgow prognostic score; mGPS, modified Glasgow prognostic score; CSS, cancer specific survival; RCC, renal cell carcinoma


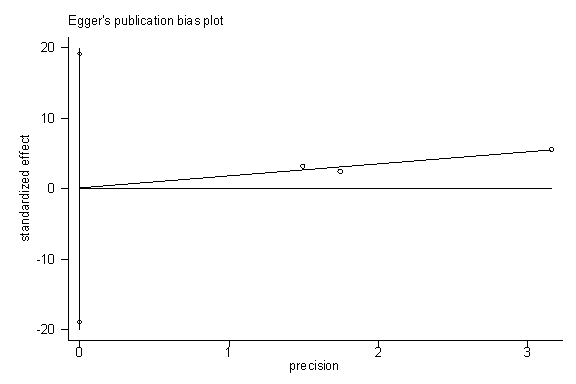

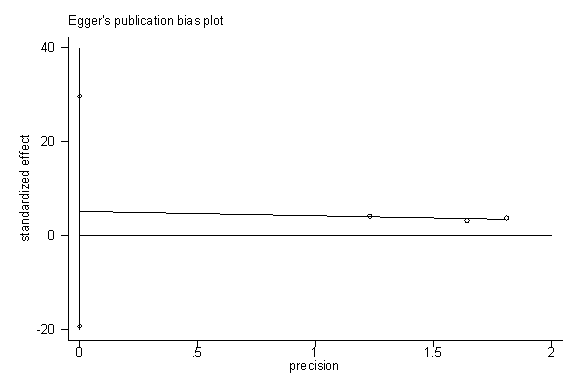


**A**

**B**

**C**

**D**

Figure S4. Egger's test and sensitivity analysis and of pooled GPS/mGPS for RFS in RCC. a, b GPS/mGPS of 1; c, d GPS/mGPS of 2

Abbreviations: CI, confidence interval; GPS, Glasgow prognostic score; mGPS, modified Glasgow prognostic score; RFS, recurrence-free survival; RCC, renal cell carcinoma

Table S1. Egger’s test result for assessing publication bias

| **outcomes** | **GPS/mGPS score of 1** | |  | **GPS/mGPS score of 2** | |
| --- | --- | --- | --- | --- | --- |
|  | *p* | 95% CI |  | *p* | 95% CI |
| OS | 0.967 | (-1.629-1.575) |  | 0.144 | (-1.078-6.021) |
| CSS | 0.536 | (1.607-2.276) |  | 0.848 | (-5.910-5.183) |
| PFS | - | - |  | - | - |
| RFS | 0.959 | (-18.929-19.122) |  | 0.228 | (-19.334-29.633) |

Abbreviations: HR, hazard ratio; CI, confidence interval; OS, overall survival; CSS, cancer-specific survival RFS, recurrence-free survival; PFS, progression-free survival; RCC, renal cell cancer; GPS, Glasgow Prognostic Score; mGPS, modified Glasgow Prognostic Score
