# Supplementary material for: A Meta-Analysis of Glasgow Prognostic Score and Modified Glasgow Prognostic Score as Biomarkers for Predicting Survival Outcome in Renal Cell Carcinoma
Source: Front Oncol. 2020 Sep 17;10:1541. doi: 10.3389/fonc.2020.01541 (PMC7527435; doi:10.3389/fonc.2020.01541)
Supplement: Supplementary file 2 [file Table_2.DOCX]

Table 1 Baseline characteristics of eligible studies

Abbreviations: NA, not available; HR, hazard ratio; CI, confidence interval; OS, overall survival; RFS, recurrence-free survival; PFS, progression-free survival; CSS, cancer-specific survival ;RCC, renal cell cancer; mRCC, metastatic renal cell cancer; ccRCC, clear cell renal cell cancer; GPS, Glasgow Prognostic Score; mGPS, modified Glasgow Prognostic Score

Table 2 Newcastle–Ottawa scale (NOS) for cohort studies quality assessment

A maximum of one star ★ for each item within the selection and outcome categories

A maximum of two stars could be given for the comparability category

Up to nine stars could be awarded

Each star ★ represented one score

Table 3 Summary of subgroup analysis results of GPS/mGPS in overall survival (OS)

Abbreviations: HR, hazard ratio; CI, confidence interval; OS, overall survival; GPS, Glasgow prognostic score; mGPS, modified Glasgow prognostic score

Figure 1 Flow diagram of literature search and selection process

Figure 2 Forest plots of pooled GPS/mGPS for OS in RCC. a GPS/mGPS of 1, b GPS/mGPS of 2

Abbreviations: HR, hazard ratio; CI, confidence interval; GPS, Glasgow prognostic score; mGPS, modified Glasgow prognostic score. OS, overall survival; RCC, renal cell carcinoma; WC institute, Winship Cancer Institute; AVAM cancer, Atlanta Veterans Administration Medical Center

Figure 3 Forest plots of pooled GPS/mGPS for CSS in RCC. a GPS/mGPS of 1, b GPS/mGPS of 2

Abbreviations: HR, hazard ratio; CI, confidence interval; GPS, Glasgow prognostic score; mGPS, modified Glasgow prognostic score; CSS, cancer specific survival; RCC, renal cell carcinoma

Figure 4 Forest plots of pooled GPS/mGPS for RFS in RCC. a GPS/mGPS of 1, b GPS/mGPS of 2

Abbreviations: HR, hazard ratio; CI, confidence interval; GPS, Glasgow prognostic score; mGPS, modified Glasgow prognostic score; RFS, recurrence-free survival; RCC, renal cell carcinoma

Figure 5 Forest plots of pooled GPS/mGPS of 2 for PFS in RCC

Abbreviations: HR, hazard ratio; CI, confidence interval; GPS, Glasgow prognostic score; mGPS, modified Glasgow prognostic score; PFS, progression-free survival; RCC, renal cell carcinoma; RCC, renal cell carcinoma
